# The Effect of Rotating Magnetic Field on Susceptibility Profile of Methicillin-Resistant *Staphylococcus aureus* Strains Exposed to Activity of Different Groups of Antibiotics

**DOI:** 10.3390/ijms222111551

**Published:** 2021-10-26

**Authors:** Marta Woroszyło, Daria Ciecholewska-Juśko, Adam Junka, Marcin Wardach, Grzegorz Chodaczek, Bartłomiej Dudek, Karol Fijałkowski

**Affiliations:** 1 Department of Microbiology and Biotechnology, Faculty of Biotechnology and Animal Husbandry, West Pomeranian University of Technology in Szczecin, Piastów 45, 70-311 Szczecin, Poland; marta.woroszylo@zut.edu.pl (M.W.); daria.ciecholewska@zut.edu.pl (D.C.-J.); 2 Department of Pharmaceutical Microbiology and Parasitology, Faculty of Pharmacy, Medical University of Wroclaw, Borowska 211a, 50-534 Wrocław, Poland; 3 Laboratory of Microbiology, Łukasiewicz Research Network—PORT Polish Center for Technology Development, 54-066 Wrocław, Poland; 4 Faculty of Electrical Engineering, West Pomeranian University of Technology in Szczecin, Sikorskiego 37, 70-313 Szczecin, Poland; marcin.wardach@zut.edu.pl; 5 Laboratory of Confocal Microscopy, Łukasiewicz Research Network—PORT Polish Center for Technology Development, Stabłowicka 147, 54-066 Wrocław, Poland; grzegorz.chodaczek@port.pl; 6 Department of Microbiology, Institute of Genetics and Microbiology, University of Wrocław, Stanisława Przybyszewskiego 63, 51-148 Wrocław, Poland; bartlomiej.dudek@uwr.edu.pl

**Keywords:** antibiotics, biofilm, β-lactam, methicillin-resistant *Staphylococcus aureus*, rotating magnetic field

## Abstract

Methicillin-resistant strains of *Staphylococcus aureus* (MRSA) have become a global issue for healthcare systems due to their resistance to most β-lactam antibiotics, frequently accompanied by resistance to other classes of antibiotics. In this work, we analyzed the impact of combined use of rotating magnetic field (RMF) with various classes of antibiotics (β-lactams, glycopeptides, macrolides, lincosamides, aminoglycosides, tetracyclines, and fluoroquinolones) against nine *S. aureus* strains (eight methicillin-resistant and one methicillin-sensitive). The results indicated that the application of RMF combined with antibiotics interfering with cell walls (particularly with the β-lactam antibiotics) translate into favorable changes in staphylococcal growth inhibition zones or in minimal inhibitory concentration values compared to the control settings, which were unexposed to RMF. As an example, the MIC value of cefoxitin was reduced in all MRSA strains by up to 42 times. Apart from the β-lactams, the reduced MIC values were also found for erythromycin, clindamycin, and tetracycline (three strains), ciprofloxacin (one strain), gentamicin (six strains), and teicoplanin (seven strains). The results obtained with the use of in vitro biofilm model confirm that the disturbances caused by RMF in the bacterial cell walls increase the effectiveness of the antibiotics towards MRSA. Because the clinical demand for new therapeutic options effective against MRSA is undisputable, the outcomes and conclusions drawn from the present study may be considered an important road into the application of magnetic fields to fight infections caused by methicillin-resistant staphylococci.

## 1. Introduction

In the last decades, antimicrobial resistance has become a significant health issue. The increasing tolerance to antibiotics is observed in a variety of bacterial species, regardless of their origin (community or clinical) [1,2]. Methicillin-resistant *Staphylococcus aureus* strains, referred to as MRSA, have acquired resistance to such β-lactam antibiotics as penicillins, cephalosporins (with the exception of ceftaroline and ceftobiprole), and to carbapenems, commonly considered antibiotics of the last resort in the treatment of hard-to-heal infections. These resistant staphylococcal strains are presently thought to comprise 25–50% of all *S. aureus* strains [3]. MRSA strains are etiological factors of more than 70,000 severe infections annually [4]. The infections caused by MRSA include a variety of disease entities, from skin, soft tissue, and wound inflammation, through alimentary and respiratory tract infections, to bone and biomaterial-related diseases [5,6]. Over time, MRSA strains have also developed resistance to other antibiotic groups, including aminoglycosides, fluoroquinolones or macrolides, thus becoming the first “super-bugs”, i.e., multidrug-resistant (MDR) pathogens [7].

Taking into account the fact of high staphylococcal adaptation to diverse physiologic niches [8], combined with the aforementioned resistance mechanism, the development of new treatment algorithms is required to prevent the increasing rate of severe infections of which MRSA is the causative agent [3]. To overcome the challenges related to staphylococcal antibiotic resistance, numerous approaches have been developed and scrutinized, though understood with moderate success, as the introduction of new molecules/treatment routes into clinical practice [9].

One of the solutions that still has not been fully investigated but is promising with regard to the matter in question is the application of various types of magnetic fields (MF) intended as an agent boosting the efficacy of antimicrobial molecules [10,11,12,13] or in the character of a self-reliant antimicrobial agent [14,15]. Our research team has long-standing experience in studying the applications of the specific type of magnetic field, referred to as the rotating magnetic field (RMF). In our previous works, we have showed that, in the case of RMF, opposite poles rotate around a certain point; therefore, the charged molecules (e.g., antibiotics) present in a medium move in an unpredictable, Brownian-type motion. Therefore, one of the results of RMF application is high mixing of the medium due to increased particle movement [16]. Moreover, in our earlier publication [10], we indicated that the combined effect of RMF and antimicrobials increases the eradication rate of *S. aureus* biofilm to 50% as compared to biofilms exposed to antimicrobials only. At that time, because our study concerned multi-cellular spatial structures, we assumed that a possible mechanism behind the observed result was related to the mixing effect caused by RMF, translating into higher penetrability of antimicrobials into the deeper layers of biofilm.

The omnidirectional and differentiated effect of RMF on micro-organisms (indicated in our earlier works [17,18]), as well as data presented, among others, by Mega-Tiber et al. (2008) [19], who showed that the application of MF may affect macromolecular synthesis and may cause protein injury in bacteria, moved us into another (the present) investigation line. The goal of this work was to analyze the influence of RMF on the changes in MRSA strains’ susceptibility to different classes of antibiotics. Our hypothesis was that RMF could have an impact on the overall antibiotic activity, resulting in a higher rate of eradication of MRSA in vitro. Although such research has never been carried out before, we assumed that at least two main factors may be responsible for such a phenomenon, namely i) the already-mentioned RMF mixing effect resulting in better transportation of antimicrobial agents; such an assumption was backed up by the study of Khoury et al. (1992), Costerton et al. (1994), and Stewart et al. (1999) [20,21,22], though performed on other types of MF and pathogenic biofilms; ii) RMF-induced alteration of staphylococcal cell functionality; this assumption was, in turn, backed up by the data shown by Golberg et al. (2014) and Alya et al. (2010) [23,24]. However, these authors also applied MFs other than RMF types in their studies.

Moreover, we assumed that other variables may have an impact on these two main possible RMF-related factors. These distinguished variables were MF characteristics, mechanism of activity of the antibiotic towards bacterial cells or the charge of the antibiotic molecule, and, last but not least, the intraspecies variability. Therefore, by using a cohesive set of analytical techniques allowing the observation and interpretation of the phenomena that were a result of the interplay of all aforementioned factors, we aimed to perform in vitro research, which could be the basis of subsequent analyses aimed at the application of RMF in the clinical practice.

## 2. Results

### 2.1. Analysis of Changes in Antibiotic Susceptibility of S. aureus Strains under the Influence of RMF

As mentioned in the methodological section, the zones of growth inhibition, as well as the MIC values for all *S. aureus* strains were measured after the end of exposure to RMF (12 h), and once more after completion of the entire incubation time (18 h—total amount of time consisting of exposure and non-exposure period). The seeded bacterial cultures on agar plates with antibiotics were subjected to the RMF for 12 h because this period allowed a well-developed bacterial lawn to be obtained, which did not change visually throughout further incubation until 18 h. Except for the bacterial growth, the inhibition zones around the antibiotic discs or E-tests were also sharp and clearly visible, so, taking both observations together, there was no reason to extend the exposure duration over the 12 h. These assumptions were reflected in further analyses in which the cultures were exposed to RMF; it was found that the results obtained immediately after RMF exposure and after further incubation without RMF did not differ regardless of the staphylococcal strain analyzed. Therefore, it can be assumed that the exposure to the RMF was long enough to obtain not only a well-developed bacterial lawn, which did not change during further incubation, but also a stable antimicrobial effect. However, it should also be noted that, based on our findings, it cannot be excluded that the time of magnetic exposure could be shorter to obtain the same or at least comparable effects, especially taking into account the different mechanisms of action of the various classes of antibiotics.

### 2.2. Disc Diffusion Test

**Figure 1:**
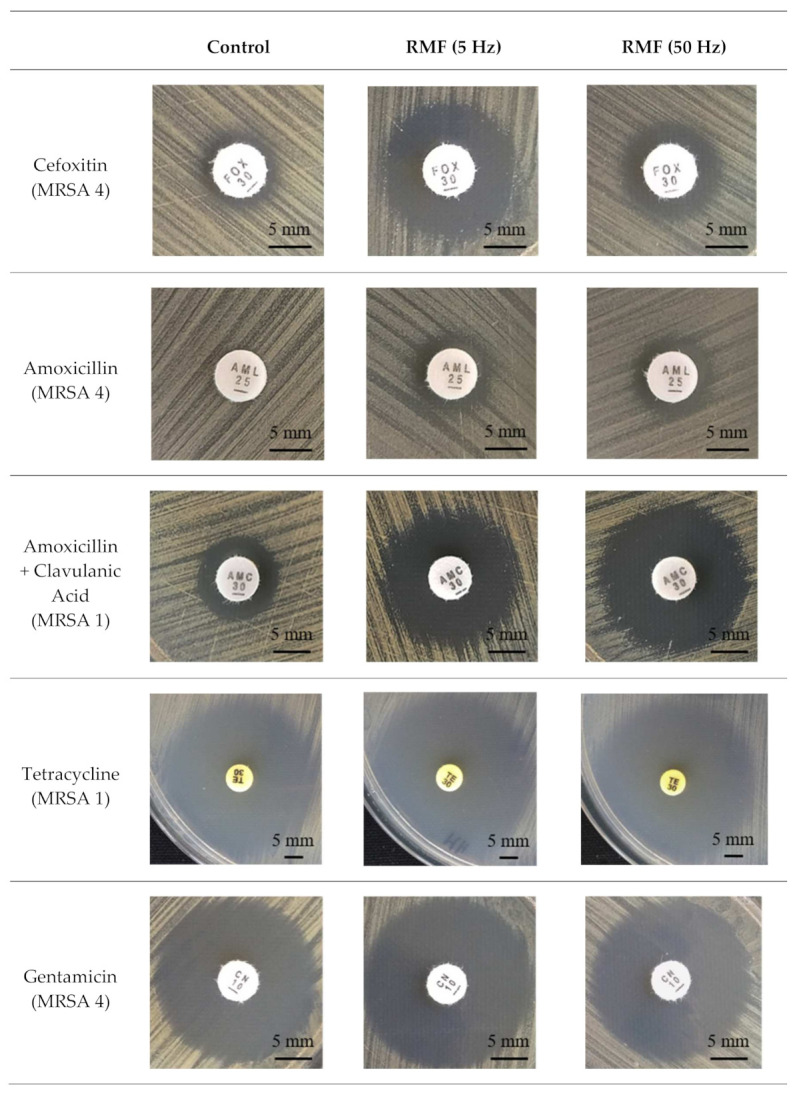

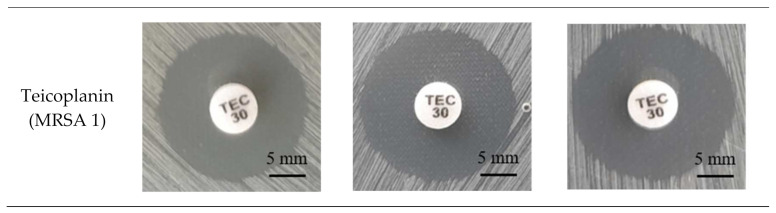
Representative pictures of growth inhibition zones (mm) in control and RMF-exposed MRSA cultures around discs with different antibiotics.

The studies showed that, according to the diameters of growth inhibition zones, all MRSA strains were characterized by increased sensitivity to cefoxitin under the influence of RMF (Table 1; Figure 1). The greatest differences and their greatest number compared to the unexposed control were found for exposure to the RMF of 5 Hz. Similar results were obtained in the analyses with amoxicillin but, in this case, RMF frequency was irrelevant for the observed changes, although the largest difference in the zone of growth inhibition compared to the control (4 mm) was obtained as a result of exposure to the RMF of 50 Hz (strains MRSA 1, 3, and 5). It is also worth noting that, for MSSA strain, no differences in changes in susceptibility to both β-lactam antibiotics were noted. Where amoxicillin was combined with clavulanic acid, the differences in the zones of growth inhibition were observed for all MRSA strains, while, in the case of the MSSA strain, the differences were again not detected. Moreover, in this case, a greater effect was observed in the cultures exposed to the RMF of 50 Hz. It was also found that the differences in the zones of growth inhibition were, in each case, substantially greater than the differences observed when amoxicillin without clavulanic acid was used. In the case of erythromycin, differences in the diameters of the zones of growth inhibition were found only for the MSSA and MRSA 7 strain (the zones were enlarged by 2 mm as compared to the control). For clindamycin, no differences were found in the zones of growth inhibition of MRSA strains, regardless of the frequency of RMF used during exposure. Only in the cultures of MSSA strain were the zones enlarged; however, only by 2 mm as compared to the controls. Similar results were obtained for ciprofloxacin, although, in this case, the lack of differences also concerned the MSSA strain. For tetracycline, differences between inhibition zones were found in the cultures of two MRSA strains (MRSA 1 and MRSA 4). The diameters of the growth inhibition zones were increased by 4 mm (MRSA 1) and 2 mm (MRSA 4) as compared to the control conditions. It can also be noted that an increase in the zones of growth inhibition was observed only in strains sensitive to this antibiotic. Most of the differences in the zones of growth inhibition under the influence of RMF, apart from antibiotics from the β-lactam group, were found for gentamicin (a representative of aminoglycosides) and teicoplanin (a representative of glycopeptides). The differences between inhibition zones around gentamicin discs were found for four MRSA strains, whereas around teicoplanin discs for five strains, including the MSSA. The diameters of inhibition zones were larger by 2–3 mm as compared to the control. Moreover, in the study with gentamicin, the differences were only observed in the cultures exposed to RMF 5 Hz.

### 2.3. Gradient MIC Strips (E-Test)

**Figure 2:**
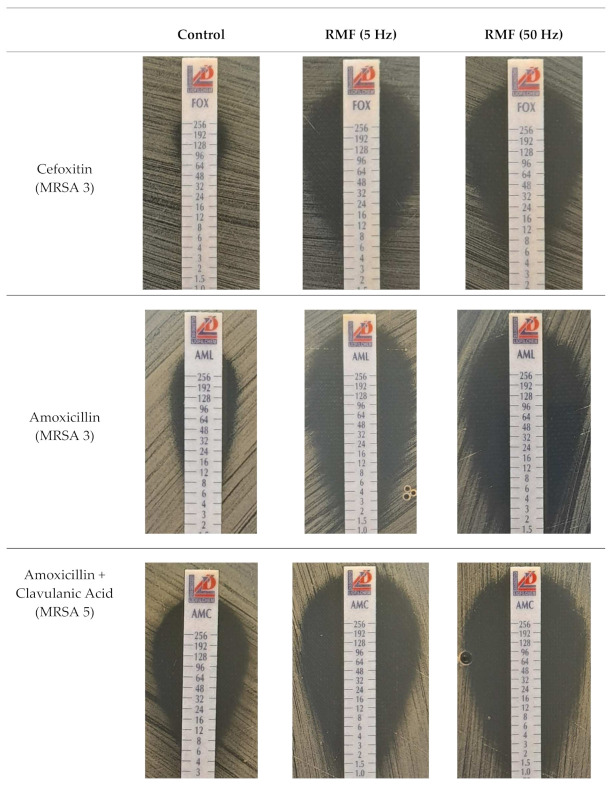

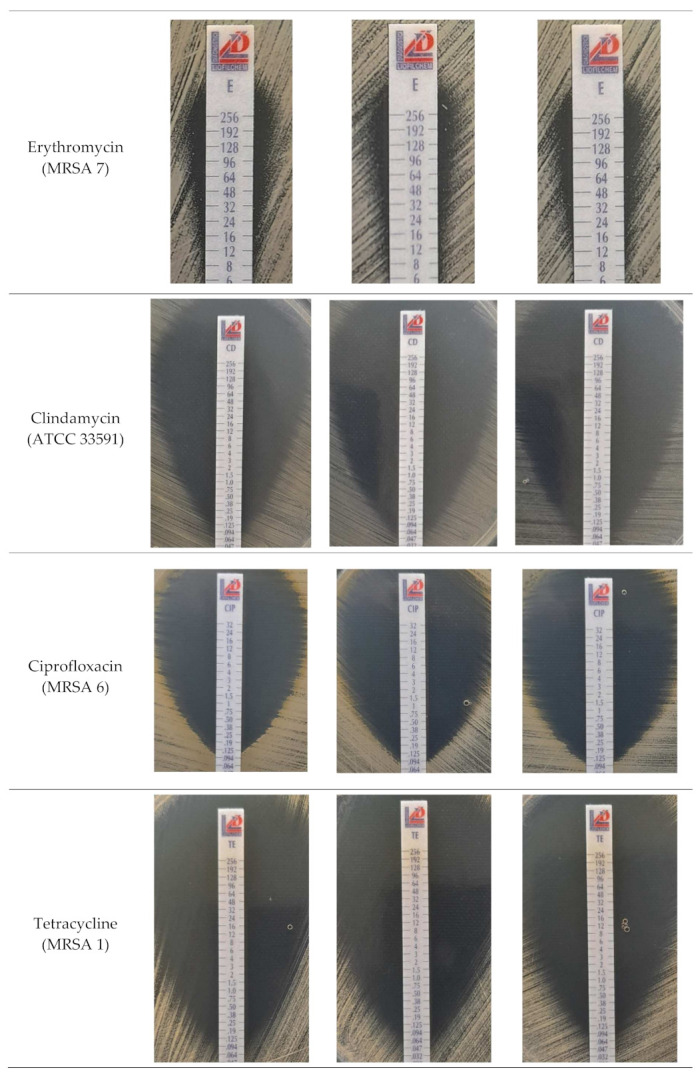

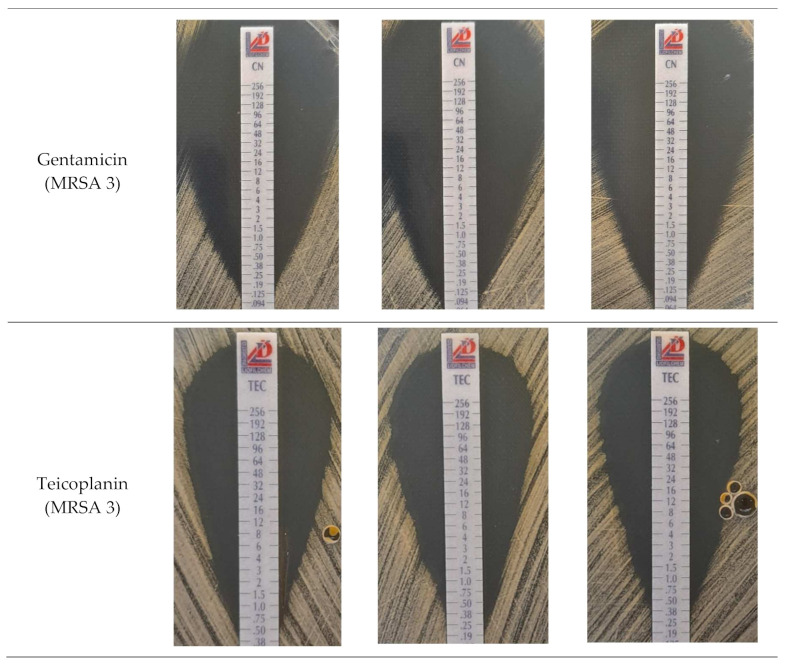
Representative pictures of gradient MIC strips (E-tests) with different antibiotics in control and RMF-exposed cultures of the MRSA strains.

It was found that in each of the RMF-exposed cultures of MRSA strains, a substantial decrease in the MIC value of β-lactam antibiotics occurred as compared to the controls (Table 2, Figure 2). In turn, no differences were found in the cultures of MSSA strains, regardless of whether cefoxitin or amoxycillin was tested. In the analyses with amoxicillin combined with clavulanic acid, differences in MIC values were also observed for all MRSA strains, while, in the case of the MSSA strain, the differences were, again, not detected. Similarly, as found in the disc diffusion tests, it was found that the differences in the MIC values were, in each case, greater than the differences observed when amoxicillin without clavulanic acid was used. The differences in MIC values of erythromycin were found in the cultures of MSSA ATCC 6538 and MRSA 7 strains exposed to the RMF of 5 Hz (these two strains were also reactive in the disc diffusion test). However, in contrast to the disc diffusion test, the use of E-test also allowed the detection of changes in the sensitivity to this antibiotic in the culture of the MRSA ATCC 33591 strain. In the case of clindamycin, lower MIC values as compared to the unexposed controls were found in the cultures of MSSA and MRSA 6 (strain not responding in the disc diffusion test) strains exposed to the RMF. In the cultures of MRSA 6 strain, the difference in MIC of clindamycin was found only when bacteria were exposed to the RMF of 50 Hz. The MRSA 6 strain was also the only one towards which the MIC value of ciprofloxacin changed; however, only when it was exposed to the RMF of 5 Hz. In the analyses with tetracycline, differences in MIC values were recorded in the cultures of MRSA 1 and MRSA 4 strains exposed to the RMF of 5 Hz (these two strains were also reactive in the disc diffusion test) and MRSA 5 strain when the RMF frequency was 50 Hz. Similarly, as found in the disc diffusion tests, most of the differences in MIC values under the influence of RMF, apart from antibiotics from the β-lactam group, were found for gentamicin (a representative of aminoglycosides) and teicoplanin (a representative of glycopeptides). The differences between MIC values of gentamicin were found for six MRSA strains, whereas, of teicoplanin, for seven strains, including the MSSA. It can also be noted that, in contrast to the disc diffusion method, the frequency of RMF was irrelevant for the observed changes in the MIC values of gentamicin.

### 2.4. Effect of RMF on Release Rate and Diffusion of Antibiotics

**Figure 3:**
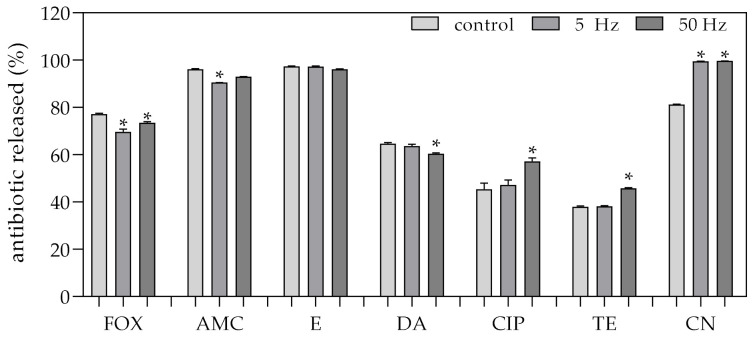
Percentage of antibiotics released from the paper discs during 120 min in control and RMF-exposed (5/50 Hz) settings. The results are presented as a mean ± SEM, calculated using six values (three from each biological replicate). * indicates statistical differences (*p* < 0.05) between control and RMF-exposed settings; FOX—cefoxitin; AMC—amoxicillin; E—erythromycin; DA—clindamycin; CIP—ciprofloxacin; TE—tetracycline; CN—gentamicin.

It was found that gentamycin, ciprofloxacin, and tetracycline were the antibiotics released from the paper disc into the agar during 120 minutes of exposure to the RMF in higher concentrations as compared to unexposed conditions (taking into account the sum of the concentrations measured in all three zones from which the agar samples were taken) (Figure 3). The obtained results concerning the concentration of those antibiotics released into the agar samples corresponded with the results showing a decrease in their concentrations in the paper discs (Supplementary Figure S1). However, the increase in the release of ciprofloxacin and tetracycline occurred only at the RMF of 50 Hz frequency, and was not observed when the samples were exposed to the RMF of 5 Hz. It is also worth noting that the difference in the concentrations of ciprofloxacin as compared to the unexposed control was determined primarily by their higher concentration in zone 3, i.e., directly under the agar disc (Supplementary Figure S1). In the case of tetracycline, an increased concentration of this antibiotic (except for zone 3) was also observed in agar samples collected from zones 1 and 2. In contrast to the aforementioned antibiotics, the significantly higher concentration of gentamycin released into the agar zones was observed regardless of the RMF frequency, but this finding was determined only by the results from zones 1 and 2 (the results obtained in zone 3 were significantly lower as compared to the control). In the case of the rest of the antibiotics included in the experiment, there was no greater release or increase in the concentration found as a result of RMF exposure as compared to the control conditions, regardless of the zone from which the agar samples were obtained or of the applied RMF frequency. On the contrary, even lower concentrations of antibiotics were detected in most of the samples cut out from the RMF-exposed agar as compared to the unexposed control.

### 2.5. Effect of RMF Coupled with β-Lactam Antibiotic on Integrity of Staphylococcal Cell Walls in the Biofilm Model

**Figure 4:**
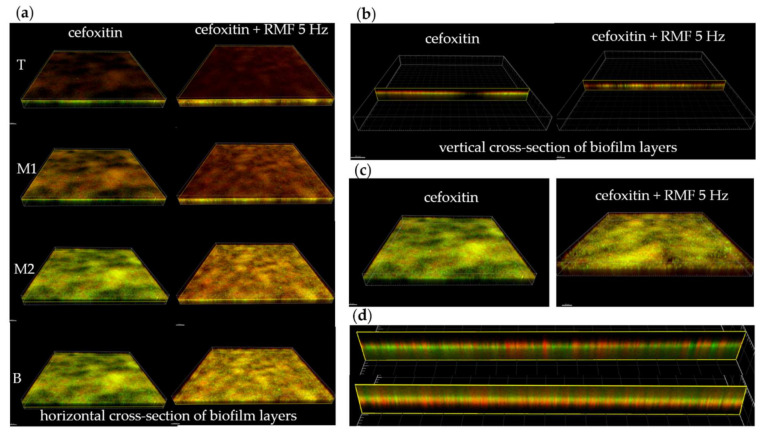
The distribution of staphylococcal cells with intact walls (green color) and with altered walls (red/orange color) in the biofilm of MRSA ATCC 33591 strain; (**a**) horizontal cross-sections of top, middle and bottom (T, M1, M2, B, respectively) layers of biofilm exposed to cefoxitin (left part) or cefoxitin coupled with the RMF of 5 Hz (right part); (**b**) vertical cross-section through biofilm layers treated with cefoxitin only (left part) or cefoxitin and RMF of 5 Hz (right part); (**c**) a stack of recorded cell layers in biofilm in settings where cefoxitin only or cefoxitin with RMF of 5 Hz were used; (**d**) distribution of cells with intact or altered walls in the biofilm unexposed to RMF and the antibiotic (upper part) and exposed to RMF of 5 Hz (lower part).

In the final investigation line, the proof-of-concept experiment, aiming to gain an insight into the potential mechanism of the observed phenomena related with increased susceptibility of staphylococci to antibiotics in the presence of RMF, was performed. Cefoxitin, a β-lactam antibiotic, was chosen for experimental purposes and introduced to biofilm (MRSA ATCC 33591 strain), which is a three-dimensional structure containing cells located in layers. The experimental setting was exposed for 12 h to the RMF of 5 Hz. After exposure, the biofilm was dyed with a combination of SYTO-9 and propidium iodide to discriminate staphylococcal cells with intact walls from cells whose walls were altered (damaged). The subsequent application of confocal microscopy allowed visualization of the extent of cell wall damage in the layers of biofilm (top, middle, and bottom ones) and compare it between the setting where cefoxitin and RMF were applied vs. the setting to which cefoxitin only was introduced (Figure 4a–c). The results showed that the number of cells with altered walls was higher in the setting where both RMF and cefoxitin were applied, as compared to the setting where only cefoxitin was used. Importantly, the comparison of biofilm unexposed to the RMF and the antibiotic to biofilm exposed to the RMF of 5 Hz frequency (Figure 4d) showed not only a higher share of wall-altered cells in the latter of the mentioned experimental settings, but also that these cells were found across the whole vertical cross-section of the biofilm. Thus, the comparison of results presented in Figure 4d (biofilm exposed to the RMF) with the results of the right side of Figure 4a (biofilm treated with cefoxitin and exposed to the RMF) may indicate that the effect displayed by RMF on staphylococci manifests itself in their cell wall alteration, similar to the effect displayed by the cefoxitin, and that the combined effects of these two agents (RMF and cefoxitin) translate into a higher rate of wall-altered staphylococcal cells.

## 3. Discussion

The study aimed to assess the possibility of application of RMF to boost the antimicrobial effect exerted by different classes of antibiotics, including β-lactams represented by cefoxitin and amoxicillin (and, additionally, amoxicillin combined with clavulanic acid); aminoglycosides represented by gentamicin; macrolides represented by erythromycin; lincosamides represented by clindamycin; quinolones represented by ciprofloxacin; tetracyclines represented by tetracycline; and glycopeptides represented by teicoplanin, against methicillin-resistant strains of *S. aureus*. The main reason for using two β-lactam antibiotics was that cefoxitin susceptibility testing is a recommended screening method for the detection of methicillin resistance in isolates of *S. aureus* [26]. In turn, application of β-lactams is frequently used together with β-lactamase inhibitors [27,28] and, for this purpose, the most standard combination of such agents (i.e., amoxicillin with clavulanic acid) was used in the present work. Therefore, the amoxicillin applied as a standalone agent served as control for the setting where both β-lactam and β-lactam inhibitor were applied.

The antibiotics selected for the study were characterized by different mechanisms of action, i.e., cefoxitin and amoxicillin—inhibition of cell wall biosynthesis in the bacteria by binding covalently to PBPs in the cytoplasmic membrane [29], gentamicin and tetracycline—inhibition or impairment of protein synthesis by blocking the 30S subunit of the bacterial ribosomes [30,31], erythromycin and clindamycin—inhibition of protein synthesis by binding to the 50S subunit of the bacterial ribosome [32], ciprofloxacin—inhibition of bacterial DNA topoisomerase and RNA gyrase [33], and teicoplanin—inhibition of synthesis of peptidoglycan by binding to amino acids (d-alanyl-d-alanine) in the cell wall [34]. Consequently, due to the aforementioned differences, the resistance mechanisms developed by micro-organisms are also of a different nature between individual antibiotics (Table 3).

It should also be noted that selected antibiotics, apart from various mechanisms of action, were also characterized by different electrical charges (Table 4). Therefore, it was possible to try to determine the impact of another variable on the observed changes (being a result of RMF activity), namely anionic, cationic, or zwitterionic character of the antimicrobials. Numerous literature data [36,37], as well as the previous experience of our research group [16], indicated that RMF influence was related to its interactions with electrically charged molecules. Therefore, it was expected that the charge of individual antibiotics may be of significance, especially taking into account the process of their diffusion in the microbiological medium. Consequently, it was assumed that the character (anionic, cationic, or zwitterionic) of the antibiotic could be one of the possible factors influencing the processes related to the rate of their release from the carrier (e.g., paper disc) and diffusion in the surrounding environment (e.g., agar medium), and thus leading to changes in the antibiotic resistance profile of the tested micro-organisms.

Furthermore, to determine whether the observed changes in antibiotic susceptibility due to the RMF exposure were strain-specific, not only the reference strains, but also seven clinical MRSA isolates were included in the study. It is well established that methicillin resistance level is not identical between staphylococcal strains [38]. For example, methicillin resistance is mediated by the expression of an altered PBP2 protein (called PBP2a) characterized by a low affinity for β-lactam antibiotics, resulting in resistance to most β-lactams [34]. However, PBP2a encoded by the *mecA* gene, which is carried on a mobile genetic element known as a staphylococcal cassette chromosome *mec* (SCC*mec*), can be regulated by two independent regulatory systems (*mecI*–*mecR*–*mecR2* and *blaI*–*blaR*) and multiple chromosomal genes [38]. Additionally, because MRSA strains, in addition to resistance to β-lactam antibiotics, can also be resistant to a number of antibiotics belonging to other classes [7], for comparison purposes, one methicillin-sensitive (lacking the *mecA* gene and showing susceptibility to methicillin in a phenotypic test with a cefoxitin-saturated disc) reference strain was used in the study. Therefore, it was possible to determine whether the changes in antibiotic sensitivity are strictly related to the methicillin resistance mechanism or may also occur in other staphylococci, regardless of their antibiotic resistance profile.

The study showed that among all antibiotic classes, the most promising results were obtained in analyses with the use of cefoxitin as a representative of β-lactams. In all MRSA cultures exposed to the RMF, noticeably larger inhibition zones around the discs with cefoxitin as compared to unexposed conditions were present. These results were also further confirmed in the study with the use of amoxicillin—the trend comparable to the one observed as a result of cefoxitin activity. It can also be seen that, although both antibiotics belong to the same class and are characterized by the same mechanism of action, they have a different charge/character [39,40] (zwitterionic vs. anionic) and, additionally, they act differently as a result of exposure to the RMF. Contrary to the effects observed, with regard to MRSA cultures, when a methicillin-sensitive strain was analyzed, no differences in the size of inhibition zones were found, regardless of whether cefoxitin or amoxicillin was used. Therefore, particularly taking into account the results obtained in the presence of cefoxitin, which is the indicator of methicillin resistance, at this stage of the experiment, it was assumed that the observed changes could be related to this specific resistance mechanism. This first assumption was additionally related to the observations from the analyses with the use of the remaining classes of antibiotics. Apart from glycopeptides represented by teicoplanin, only a few differences in the sizes of inhibition zones around the discs with erythromycin (2), clindamycin (1), tetracycline (2), ciprofloxacin (0), and gentamicin (4) between RMF-exposed and control cultures were observed (Table 1). Moreover, the differences were noticeably smaller as compared to β-lactams and, in any case, did not exceed 4 mm. Furthermore, no correlation between the charge of the antibiotic and the observed effect was found. Importantly, the mechanism of action of none of the above-mentioned antibiotics involves the disturbance of cell wall structure or synthesis (Table 3). In contrast, in the case of teicoplanin, whose mechanism of action relies on the inhibition of cell wall synthesis [41] (and, thus, with regard to the site of activity and the effect, resembles the activity of β-lactam antibiotics), a change in inhibition zone diameters was observed in cultures of five strains exposed to the RMF (Table 1). The differences were not as large as in the case of β-lactam antibiotics (they did not exceed 2 mm in comparison to the control). However, it should be noted that vancomycin-resistant strains were not used in the studies and, thus, even in the control cultures, the zones of growth inhibition were relatively large. In this context, in the case of β-lactams applied against MSSA, the differences in the inhibition zones were not visible at all.

The next part of the study aimed to determine whether the observed changes (or their lack) in sensitivity to antibiotics caused as a result of the RMF exposure can be related to the specific concentration of antimicrobial used in the disc diffusion test. It was assumed that a too low concentration of an antibiotic could be a possible reason for the lack of differences in the zones of growth inhibition found in the cultures of MSSA strains or the remaining MRSA when antibiotics other than β-lactams were applied. The results of this part of the study confirmed that the lack of the influence of the RMF observed in some of the cultures tested with erythromycin, clindamycin, tetracycline, ciprofloxacin, gentamicin, as well as teicoplanin using the disc diffusion assay could be related to a relatively low concentration of these antimicrobials in the carrier. Although this observation did not apply to all strains, at least in some of the cultures, reduced MIC values were observed in comparison to unexposed controls (although no changes in the diameters of inhibition zones were found in the disc diffusion method). Nevertheless, the exposure to RMF did not change the susceptibility level of the MSSA ATCC 6538 strain to both β-lactams included in the experiment, even when the E-tests were used.

Apart from the aforementioned analyses of different groups of antibiotics, the current research also included the use of a β-lactam antibiotic (amoxicillin) combined with an inhibitor of β-lactamases, referred to as clavulanic acid. This part of the study was carried out to potentially elucidate the fact of the relatively small (as compared to cefoxitin) differences in inhibition zones for amoxicillin observed in the cultures of RMF-exposed and control staphylococcal strains. As was demonstrated by Harrison et al. (2019) [42], such β-lactamase inhibitors as clavulanic acid may find potential application in the eradication of infections caused by MRSA strains thanks to the property of clavulanic acid manifesting itself in an increase in the affinity of PBP2a for β-lactams. For these reasons, we assumed that the use of β-lactam in combination with β-lactamase inhibitor may provide a good model to determine whether the changes in the sensitivity of MRSA strains, observed under the influence of RMF, are related to the mechanism of action of β-lactam antibiotics. First of all, the results obtained under control conditions allowed one strain (MRSA 2) insensitive to the presence of clavulanic acid (the use of amoxicillin in combination with clavulanic acid did not affect its growth inhibition zones as compared to the test with amoxicillin only) to be selected (Table 1). For the remaining strains, the zones of growth inhibition were enlarged due to the presence of clavulanic acid and, hence, all these strains were defined as sensitive to the presence of β-lactamase inhibitors. It was also found that, due to the RMF exposure, the differences in the zones of growth inhibition were, in each case, greater than the differences observed when amoxicillin without clavulanic acid was used. However, in the case of the MSSA strain, the differences were, again, not detected. This finding indicates that the observed changes under the influence of RMF may be related to the mechanism of resistance to β-lactam antibiotics. Moreover, it can be noted that the differences were also found in the case of the culture of the MRSA 2 strain for which the effect of clavulanic acid was not detected in the control cultures. This offers the potential for further use of clavulanic acid, not only in the case of MRSA susceptible to its presence (to further boost the effect caused by the β-lactamase inhibitor), but also in the case of resistant strains.

**Figure 5:**
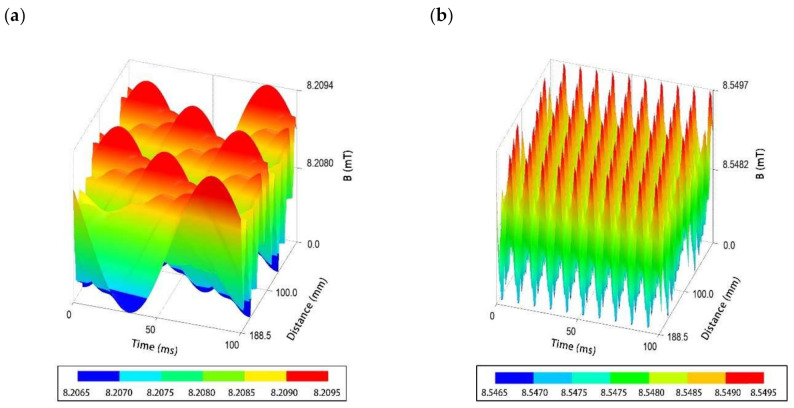
Changes in magnetic flux characteristic depending on the applied AC frequency: (**a**) 5 Hz; (**b**) 50 Hz.

The research previously conducted by our research group [17,18,43], as well as the studies of several other authors [36,44,45], revealed that the strength of MF impact (regardless of its type or the phenomenon analyzed in its presence) depends also on the intensity and/or the frequency of MF, because these two factors determine the physical characteristics of the magnetic signal [46,47]. Therefore, the potential impact of these variables was also analyzed with regard to the observed changes in the antibiotic susceptibility profiles between the analyzed strains. In the case of the RMF set-up used in the present study, the frequency of AC determines the MF intensity and, importantly, it is responsible for the physical characteristics of the magnetic wave shape. For this reason, the analyses including the AC frequencies of 5 and 50 Hz at which the RMF was generated allowed an MF of different parameters to be obtained. As shown by the simulation calculations, at 5 Hz (https://www.youtube.com/watch?v=2dxP7nzEThA accessed on 18 October 2021), the amplitude of the RMF was characterized by a longer period between magnetic induction maximal strength state (100 ms with *B*_max_ 8.1 mT) as compared to the RMF generated at 50 Hz (the highest current frequency in the applied set-up). The RMF generated at 50 Hz (https://www.youtube.com/watch?v=4xggMktw3ho accessed on 18 October 2021) was characterized by a shorter period, with 10 ms between magnetic induction maximal strength state with *B*_max_ 8.5 mT (Figure 5; Supplementary Figure S3).

Simultaneously, the applied AC frequencies generated magnetic flux rotation around the stator with different synchronous speeds of 150 rpm and 1500 rpm, respectively (calculations performed on the basis on the manufacturer characteristics of the stators). In line with our assumptions, it was shown that the results obtained during the analysis using the disc diffusion method and E-tests were determined by the characteristics of the generated RMF. For example, it can be seen that, for cefoxitin and gentamicin, the diameters of the zones of growth inhibition were larger as a result of the exposure to RMF of 5 Hz, compared to RMF of 50 Hz. Similar trends were found in the analyses with E-tests—although, in the cultures of the three strains, the MIC did not differ depending on the RMF frequency, the remaining strains were characterized by lower MIC values as a result of exposure to the RMF of 5 Hz. On the other hand, in the case of amoxicillin alone or coupled with clavulanic acid, the tendency was the opposite, which means that RMF of 50 Hz was more effective. In turn, in the analyses with teicoplanin, the changes occurred regardless of the frequency used. For the remaining antibiotics, no recurring trend could be found due to the relatively low number of RMF-reactive cultures. Nevertheless, due to the lack of connection of the obtained results with the charge/character and the mechanism of action of individual antibiotics, at this stage, apart from noting their presence, it is impossible to explain their nature. However, the obtained results allow the conclusion that the frequency of the generated RMF should be adjusted primarily to a specific antibiotic (not only with regard to its class), and also, although to a lesser extent, individually to each bacterial strain/isolate.

Summarizing the previous stages of the analyses, the most promising results related to the possibility of using RMF to change the antibiotic resistance of MRSA strains concern two groups of antibiotics, β-lactams and glycopeptides, the common feature of which is the site of their antimicrobial activity, i.e., the bacterial cell wall. We are aware that, although the general mechanism of action of β-lactam antibiotics is similar, there are some differences in their specific activity, e.g., related to different binding sites with the PBP2a protein or to the binding energy value [38,42]. Similarly, in a group of glycopeptide antibiotics, the relationship between vancomycin and teicoplanin minimum inhibitory concentration (MIC) is not clearly defined [48,49]. Despite some correlation between vancomycin and teicoplanin MIC for *S. aureus*, the reports have highlighted the importance of both species and strain-specific MIC differences and that the microbiological activity of the two compounds cannot be considered to be unequivocally equal [50]. For this reason, at this stage, we are not able to conclude that our results would apply to the whole group of β-lactams or glycopeptides. Nevertheless, the positive results obtained in the present study encourage us to perform the next experiments, in which the findings of the present study will be investigated further by, among others, analyzing a higher number of representatives of different groups of antibiotics, different modes of bacterial exposures to the RMF, as well as optimization of exposure duration.

The next line of investigation was performed to analyze another crucial variable in the applied experimental system, namely the differences in antibiotics’ release and diffusion, which may potentially occur between bacterial cultures exposed and unexposed to the RMF. Considering our previously published data regarding, e.g., mixing efficiency under the influence of the RMF [16,51], it can be noticed that MF influence was related to its interactions with electrically charged molecules. Therefore, we aimed to investigate whether magnetic exposure alters the release of the antibiotics from the paper discs and their diffusion in the agar medium. Moreover, the study aimed to determine whether the effect of applying RMF is dependent on the charge of the antibiotic molecules (Table 4) and correlated with the results of the changes in the antibiotic sensitivity observed in the biological study (Table 1 and Table 2).

Although gentamycin, ciprofloxacin and tetracycline were the antibiotics released from the paper disc into the agar during 120 minutes of exposure to the RMF in higher concentrations as compared to unexposed conditions, in the case of ciprofloxacin, it did not translate into changes in the antibiotic susceptibility profile with regard to any staphylococcal strain. In the case of tetracycline, the changes were found only in two staphylococcal strains. On the other hand, the difference in the concentration of ciprofloxacin (in the RMF-exposed setting as compared to unexposed control) was determined primarily by its higher concentration in zone 3, i.e., directly under the agar disc. It could not have determined the assumed effects in the disc diffusion method and E-tests. It is also worth noting that a significantly higher concentration of gentamycin released into the agar zones was observed regardless of the applied RMF frequency, while, in the case of diffusion tests, more differences (in inhibition zones and MIC values) were found under the influence of RMF of 5 Hz. It should also be noted that LC-MS/MS analyses showed lower concentrations of β-lactams (antibiotics for which the most significant changes under the influence of RMF were found) in the analyzed zones of agar samples as compared to the unexposed control.

The above observations provide a strong premise that the observed effect of changes in the susceptibility of staphylococcal strains is not related to the direct impact of RMF on the particles of these antibiotics’ molecules (at least not in the applied diffusion tests). If this is the case, the RMF must react directly with the staphylococcal cells (of MRSA strains, particularly). Indeed, the team of Oncul et al. (2016) [52] indicated that time-varying MFs may interact with the physicochemical potential of such microbial cells’ external structures as cell membranes and walls, leading to their physiological alterations and changes in the level of formed free radicals. Following this lead, we performed a proof-of-concept experiment in which we exposed the biofilm of MRSA to β-lactam antibiotic (cefoxitin), coupled or not with RMF of 5 Hz frequency. The untreated biofilm of the same strain and the biofilm exposed to RMF only served as control settings in this experiment. The images of cell-wall-compromised and intact biofilm-forming cells were captured in *x*, *y*, and *z* axis and visualized using image processing software. The rationale behind using a biofilm in vitro model for this experiment was the fact that this spatial microbial community is a three-dimensional structure, consisting of layers formed by immobilized cells [53]. Thus, we made use of biofilm as the cells’ carrier. Such an approach allowed us to omit the challenges related to the application of other immobilizers (various types of hydrogels) whose presence could be another variable, potentially affecting cell walls’ integrity. We hypothesized that, because RMF is the factor of wave-like and not of corpuscular properties [54], its activity against staphylococci should not be hindered by such physical obstacles as biofilm height or the cellular density within particular layers. The above-mentioned features are considered the major factors (together with the presence of extracellular matrix) impeding the activity of antibiotics targeted against biofilm [55]. Our primary observation (Figure 4) was that the structure of biofilm unexposed to RMF and/or antibiotics contains a certain number of cells of altered wall integrity, mostly in the top and bottom parts of its structure. The highest cellular density (and the highest number of cells with intact cell walls) was observed in the middle part of the biofilm. The horizontal and vertical cross-sections (of ~2µm thickness) of biofilm treated with cefoxitin revealed that this antibiotic was able to disintegrate the walls of cells located within the top and upper-middle layer of the biofilm. At the same time, the lower-middle and bottom layers remained mostly intact. Such observation is consistent with the generally accepted statement of highly elevated tolerance of biofilms against antibiotics [55,56,57]. In turn, when cefoxitin was coupled with RMF of 5 Hz frequency, a high number of wall-compromised cells was observed within the top and upper-middle, but also in the lower-middle and in the bottom layers of the biofilm. The question that should, thus, once again be addressed is whether this observed, increased number of wall-compromised cells (regardless of their spatial position in biofilm) is a result of increased penetrability of cefoxitin (caused by the RMF presence) through biofilm layers or the observed effect is induced by the RMF itself (directly). The data, already presented in this work, show no increased diffusion of β-lactams in agar medium in the presence of RMF, although we are aware of potential biases related with comparisons of results obtained from different methodological approaches. In turn, a direct comparison of unexposed biofilm to biofilm exposed to RMF (Figure 4d, upper and lower part, respectively) shows a higher number of wall-compromised cells in the later setting, regardless of their position (height) in the biofilm structure. Such observation, if further confirmed on a larger number of strains, matches the wave properties of magnetic field [58], which is not constrained by the already-mentioned physical factors constituting significant impediments for antimicrobial molecules applied against biofilm. At this moment, we cannot conclusively exclude the possibility of increased permeability of antibiotics through the biofilm layers in the presence of RMF because the different models of biofilms cultured in vitro may display various characteristics translating into different results in this regard [59]. Nevertheless, analyzing data obtained from the biofilm model applied in this particular study, we may conclude that the increased susceptibility of MRSA cells to β-lactam antibiotic is caused by the activity of RMF, affecting the structure of staphylococcal cell walls (which are also the target size of cefoxitin). Undoubtedly, further research is required to determine whether the observed effect is of additive or synergistic character. Nevertheless, this observation brings us closer to the nature of this effect, as it allows focus on the particular component of the staphylococcal cell, namely the cell wall.

The goal of the present research was to analyze the effect of rotating magnetic field on the susceptibility profile of methicillin-resistant *S. aureus* strains to different groups of antibiotics. This seemingly easy-to-perform analysis was thus planned as consisting of barely three main variables, namely RMF, staphylococcal strains, and antibiotics. However, within the course of the study, these variables developed into a subsequent high number of factors, all crucial with regard to the matters analyzed. First of all, it occurred that RMF specifics should be calibrated thoroughly to obtain repeatable conditions of exposure. Based on our previous experience [18], two frequencies (5 and 50 Hz) were chosen. The second major variable, the staphylococcal strains, involved many more aspects to take into account. The significance of intraspecies variability is presently more and more stressed, especially with regard to studies of antimicrobial efficacy [60]. Therefore, we decided to analyze the impact of antibiotics and RMF on not only the reference staphylococcal strain, but we also included clinical isolates to the experiment. Indeed, we observed a spectrum of answers to the same antimicrobial (coupled with the RMF), depending on which staphylococcal strain it was applied against. This phenomenon, of pivotal significance in the studies on MF impact on micro-organisms, is often neglected, i.e., only one reference strain as an example of a given species is analyzed [61,62,63]. This specific methodological approach requires a separate line of investigation if proper conclusions on the observed effects are to be drawn. Moreover, although the experimental group consisted of staphylococcal strains of methicillin resistance pattern (and single MSSA strain provided as a control strain), it should be once more reminded that this specific resistance mechanism is conditioned by different binding sites and energy value of β-lactam antibiotics to altered PBP proteins [38,42]. Thus, the effects, after exposure to the RMF and the antibiotic, were expected to differ as well; the estimation of the impact of the above-mentioned factors related to binding of β-lactam to the cell wall is presently beyond the scope of this research. The other variable, related to both the micro-organisms and the antimicrobials, was the experimental setting. From the macro-perspective, the applied agar plate should be considered a semi-solid surface. From the micro-perspective, the hydrocolloid agar, in which diffusion of antibiotics from antibiotic-containing disc or E-test occurs, consists in ~98% of water. While the agarose, which represents approximately two-thirds of the natural agar composition is of neutral charge, the remaining one-third of agar consists of agaropectin, which is negatively charged due to the presence of pyruvate and sulfate groups [64]. The agar polymer forms mesh-like structures of pore size inversely proportional to the concentration of the agar (approximately 70 nm radius in the case of agar applied in this study) [65]. Presently, there are no data conclusively indicating a correlation between the pace of diffusion of the specific antibiotic through such negatively charged, water-filled nano-pores of agar and the antibiotic molecular mass, hydrophobicity, electric charge, and concentration. Only general assumptions on these aspects can be made, while the details, of potentially high significance, are still obscure. The above-mentioned remarks were presented to highlight the fact that the number of unknowns and variables revealed during the performed studies was increasing, along with the number of techniques performed and the amount of data collected.

Nevertheless, the outcomes of this study indicate explicitly that the major component standing behind the increased susceptibility of methicillin-resistant staphylococci to antibiotics is these microbes’ cell wall. This statement can be elucidated not only from the fact that the application of antibiotics for which the cell wall is the target site correlated with the largest inhibition zones of staphylococcal growth (although their sizes were modified by strain-dependent variability) in the presence of RMF, but also from the fact that these changes were observed in MRSA but not in MSSA strains. Having indirectly proven that RMF’s mechanism of action is related to staphylococcal cell wall, we confirmed it directly by microscopic observation of a high number of cells with altered walls as a result of exposure to the RMF.

We believe that our findings significantly narrow the number of possible drawbacks related to the application of MFs against bacterial infections, such as, in particular, the yet unresolved mechanism of interaction. The data presented in this research are another premise that future studies on the mechanism of RMF impact on staphylococci should be focused on the interaction of MF with the septation machinery correlated to peptidoglycan and/or the peptidoglycan-modifying enzymes [13].

From the clinical perspective, it should be stated explicitly that our observations may be applied only to the RMF-responsive strains of methicillin-resistant *S. aureus* (due to the intraspecies variability, some strains may not react to exposure to RMF) and/or to the specific antibiotic used. Nevertheless, it is reasonable to speculate that the application of RMF coupled with β-lactams may, at some point, become an attractive treatment option with regard to infections caused by recurring staphylococcal strains, as is the case in chronic bone infections [66]. Nevertheless, before such treatment can be applied, the mechanism standing behind the impact of MFs needs to be fully elucidated. We believe that the high amount of data presented in this work, pointing to the staphylococcal cell wall as the primary target of RMF action, allows to ask the final question, namely how the RMF affects the staphylococcal cell wall structure. Therefore, this work may be considered an important step into the application of MFs in future clinical practice to fight staphylococcal-based infections.

## 4. Materials and Methods

### 4.1. Microorganisms

Two reference staphylococcal strains, including one MRSA—American Type Culture Collection (ATCC 33591) and one MSSA (ATCC 6538), and seven clinical MRSA isolates (MRSA 1—MRSA 7) were used for experimental purposes. All analyzed clinical isolates were provided from the Strain Collection of the Department of Pharmaceutical Microbiology and Parasitology of Wroclaw Medical University. All these strains were previously isolated from chronic wounds of patients treated in the Teaching Hospital of Wroclaw Medical University (Wroclaw, Poland) during another project, approved by the Bioethical Committee of Wroclaw Medical University, protocol # 8/2016. The strains’ species affiliation was confirmed using the automated Becton-Dickinson Phoenix 100 system (Franklin Lakes, NJ, USA), whereas methicillin resistance was measured according to the guidelines of the European Committee on Antimicrobial Susceptibility Testing (EUCAST, 2021) [67] using the disc diffusion method with cefoxitin antibiotic (30 µg), as well as by detection of the *mecA* gene using primers and PCR conditions previously described by Oliveira and de Lencastre (2002) [68].

### 4.2. Rotating Magnetic Field Generator

**Figure 6:**
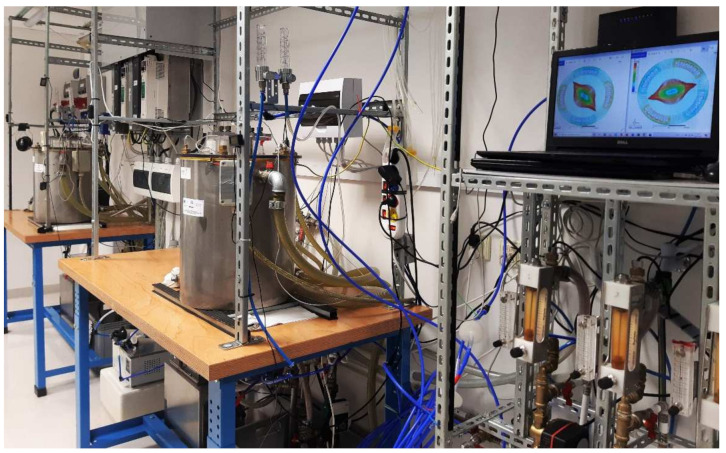
RMF generator with monitoring and control equipment.

The core of the RMF bioreactor (Figure 6) was a 3-phase, 4-pole stator with an internal diameter of 16 cm and height of 20 cm, consisting of 12 groups of three coil sets [69]. The alternating current (AC) frequency supplied to the RMF generator was regulated using the Unidrive M200 inverter (Control Techniques, Nidec Industrial Automation, Poznan, Poland). The temperature in the RMF reactor chamber was controlled using a water-fed cooling/heating system equipped with several temperature probes with sampling deviation in the accuracy range ±1.0 °C. The correct temperature distribution in the RMF bioreactor was maintained by air flow provided during exposure (2 L/min, 35 °C, RH 60%). The distribution of magnetic induction (*B*) in the reactor chamber was performed at an initial voltage of AC of 100 V and AC frequencies of 5 Hz and 50 Hz using the Ansys Maxwell simulation software ver.19.1 (ANSYS, Inc., Canonsburg, PA, USA) and using a tesla meter (SMS-102, Asonik, Tuczno, Poland).

### 4.3. Analysis of the Impact of RMF on Changes in Antibiotic Susceptibility

#### 4.3.1. Disc Diffusion Method

In the first stage of the study, the impact of exposure to the RMF generated at AC frequencies of 5 and 50 Hz on the changes in susceptibility to different classes of antibiotics, including β-lactams, aminoglycosides, macrolides, lincosamides, quinolones, and glycopeptides, was analyzed.

Additionally, analyses of the influence of RMF on changes in bacterial strain sensitivity to antibiotics in combination with an inhibitor of β-lactamase enzymes were performed. A commonly used variant of active substances (amoxicillin with clavulanic acid) was used.

The antibiotic susceptibility assessment was performed according to EUCAST guidelines (2021) [67]. The bacterial cultures were adjusted to 0.5 McFarland standard, which corresponds to 1–2 × 10^8^ CFU/mL, and spread evenly over the surface of M–H agar (Graso Biotech, Jablowo, Poland) plates using sterile cotton swabs. After the application of antimicrobial discs on the M–H plates, the bacteria were exposed to the RMF for 12 h. After completion of the exposure time, the cultures with the antibiotic discs were taken out from the RMF generator and incubated at 35 ± 1 °C without RMF until 18 h of incubation were completed (total amount of time consisting of exposure and non-exposure period). The zones of growth inhibition were measured after the end of exposure to RMF, and once more after completion of entire incubation time.

The same bacterial cultures, incubated under the same conditions but without exposure to the RMF, were used as a control setting. Both in the RMF generator and the incubator, the same temperature (35 ± 1 °C) and relative humidity RH (60%) were maintained throughout the entire experiment.

The following antibiotic discs were used: cefoxitin (30 μg/disc), amoxicillin (25 µg/disc), amoxicillin/clavulanic acid (20/10 μg/disc), erythromycin (15 μg/disc), clindamycin (2 μg/disc), ciprofloxacin (5 μg/disc), gentamycin (2 μg/disc), tetracycline (30 μg/disc), and teicoplanin (30 μg/disc) (Oxoid, Basingstoke, UK).

#### 4.3.2. Gradient MIC Strips (E-Test)

Because the disc diffusion method only allows the analysis of the impact of a single concentration of an antibiotic, in the second stage of the experiment, the E-test strips with exponentially decreasing antibiotic concentrations were applied for all of the antibiotics that were previously used in the disc diffusion test.

The cultures with the E-tests were exposed and incubated in the same way as described for the disc diffusion method. The E-tests containing cefoxitin, amoxicillin, amoxicillin/clavulanic acid, erythromycin, clindamycin, tetracycline, ciprofloxacin, gentamicin, and teicoplanin were obtained from Liofilchem (Roseto degli Abruzzi, Italy). The analyses were performed on M–H agar in accordance with the E-test manufacturer’s recommendations.

**Figure 7:**
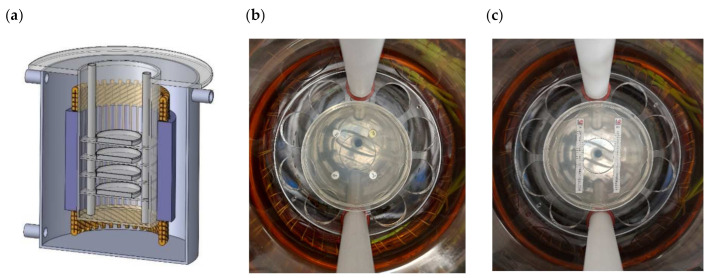
Location of Petri dishes (**a**) with antibiotic discs (**b**) and E-tests (**c**) in RMF bioreactor.

The location of the Petri dishes in the RMF reactor chamber and the location of antibiotic discs and E-tests in Petri dishes are presented in Figure 7.

### 4.4. Analysis of the Impact of RMF on the Diffusion of Antibiotics in the Agar Medium

**Figure 8:**
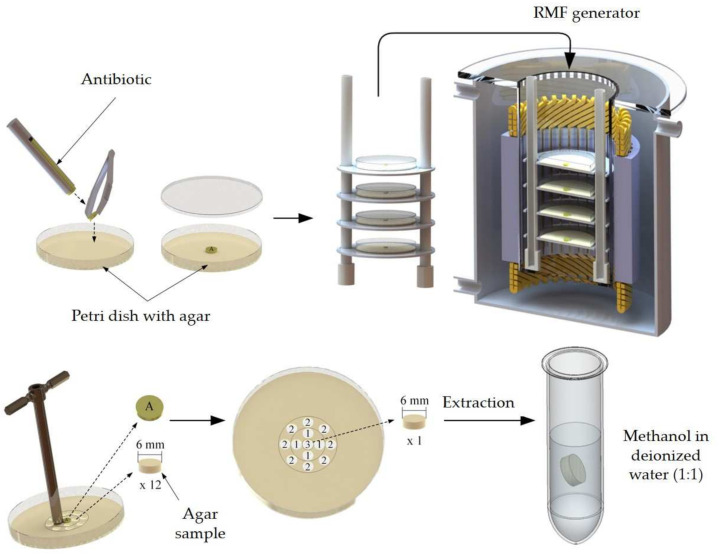
Schematic presentation of the analysis of the impact of RMF on the diffusion of antibiotics in the agar medium. 1—agar sample from the proximal zone (zone 1); 2—agar sample from the distal zone (zone 2); 3—agar sample under the antibiotic paper disc (zone 3); A—antibiotic paper disc.

In order to analyze the impact of the RMF on antibiotic diffusion, the same discs as the ones used for the analysis of antibiotic resistance were placed on Petri dishes with 1.7% agar (Graso Biotech, Jablowo, Poland) (agar concentration was equal to the concentration of M–H agar applied in previous experiments) and exposed to the RMF generated at 5 and 50 Hz. After 120 min, the plates were removed from the RMF generator, the paper discs with antibiotics were removed, and, using a cork borer, cylindrical agar samples 6 mm in diameter were cut out (4 samples from the proximal zone (zone 1), 8 samples from the distal zone (zone 2), representing 50% of the total agar volume in each zone) and, additionally, 1 sample was obtained from the agar where the antibiotic disc was placed (zone 3) (Figure 8). To extract the antibiotic, the paper discs and agar samples were placed in 0.5 mL of methanol (Stanlab, Lublin, Poland) in deionized water (1:1) and incubated with shaking (250 rpm; Biosan, Riga, Latvia) for 3 h. Next, the methanol–water mixtures with the extracted antibiotics were filtered through a syringe filter (0.22 µm pore diameter) and analyzed by liquid chromatography–tandem mass spectrometry (LC–MS/MS) technique (1260 Infinity II Series Liquid Chromatograph, Agilent, Santa Clara, CA, USA). An InfinityLab Poroshell 120 EC-C18 column (Agilent, Santa Clara, CA, USA) with a particle diameter of 2.7 µm equipped with a guard column was used for chromatographic separation. A mass spectrometer (Ultivo G6465B, Agilent, Santa Clara, CA, USA) coupled to the chromatograph was used to detect and identify the assessed antibiotics. The quantitative analysis was based on calibration curves prepared with the use of antibiotic standards (Millipore Sigma, St. Louis, MO, USA). The results were converted and presented as the concentration of antibiotic remaining in the disc and released to each zone.

### 4.5. Visualization of the Impact of RMF and β-Lactam Antibiotic on the Integrity of Staphylococcal Cell Walls

Initially, MRSA reference strain (ATCC 33591) was plated onto Columbia agar with 5% sheep blood (Graso Biotech, Jablowo, Poland) and cultivated for 24 h at 37 °C. After incubation, one colony-forming unit (CFU) was transferred into 10 mL of tryptic soy broth (TSB, Graso Biotech, Jablowo, Poland) and incubated another 24 h at 37 °C with shaking (200 rpm). Next, cultures were diluted in TSB supplemented with 1% glucose to obtain bacterial suspension equal to 1 × 10^5^ CFU/mL. In the next step, 1 mL of the bacterial suspension was added to a 24-well plate (1 mL into each well), (VWR, Radnor, PA, USA). To obtain biofilm, the plates with bacterial suspension were incubated for 48 h at 37 °C.

Prior to the addition of the β-lactam antibiotic (cefoxitin, Pol-Aura, Olsztyn, Poland), the TSB medium was removed and the wells with biofilm washed with PBS buffer. Then, 200 µL of PBS containing cefoxitin (8 mg/mL) was added to each well of a 24-well plate. As it was determined in the initial step of the study, such a concentration of cefoxitin caused approximately a 50% reduction in growth of biofilm-forming bacteria. After the application of antimicrobial, the biofilms were exposed to the RMF of 5 Hz for 12 h at 36 ± 1 °C.

The MRSA ATCC 33591 biofilms exposed to the RMF only or unexposed to any of the investigated factors (RMF or cefoxitin) served as control settings.

After the above-mentioned procedures, the medium from biofilm-containing wells of 24-well plates was removed and replaced with 200 µL of Filmtracer™ LIVE/DEAD™ Biofilm Viability Kit (Invitrogen, Thermo Fisher Scientific, Eugene, OR, USA) solution and incubated at room temperature for 15 min. After incubation, the solution was removed and the wells were gently rinsed 3 times with sterile water. Next, the water was removed. The biofilms were analyzed using a confocal microscope Leica SP8 (Wetzlar, Germany) with a 25× water dipping objective using 488 nm laser line and 500–530 nm emission to visualize SYTO-9 and 552 nm laser line and 575–627 nm emission to visualize propidium iodide (PI), in a sequential mode. Images are maximum intensity projections obtained from confocal Z stacks with ~2 µm spacing in Z dimension. PI is represented in red/orange and SYTO-9 in green color. The obtained biofilm images were further analyzed using Imaris 9 (Abingdon, UK) software.

### 4.6. Statistical Analysis

The data obtained in this study concerning the changes in antibiotic concentrations in paper discs and agar samples in the control and RMF-exposed settings were presented as means ± standard errors of the means (SEM) obtained from three different measurements (plus technical repetitions). Statistical differences between RMF-exposed and control, unexposed settings were determined by one-way analysis of variance (ANOVA) and Tukey’s post hoc test. Differences were considered significant at a level of p<0.05. The statistical analyses were conducted using GraphPad Prism 9.0 (GraphPad Software Inc., San Diego, CA, USA).

## Figures and Tables

**Table 1:** Growth inhibition zones (mm) of RMF-exposed staphylococcal strains around antibiotic discs.

Culture Conditions	Staphylococcal Strain								
	ATTC 33591	ATTC 6538	1	2	3	4	5	6	7
	Cefoxitin—β-Lactams—Methicillin Resistance Indicator								
Control	12	27	6	7	6	7	9	13	6
RMF (5 Hz)	18 ^a^	27	16 ^a^	21 ^a^	18 ^a^	16 ^a^	15 ^a^	16 ^a^	16 ^a^
RMF (50 Hz)	15 ^b^	27	14 ^b^	15 ^b^	9 ^b^	11 ^b^	10 ^b^	13 ^b^	13 ^b^
Amoxicillin—β-lactams									
Control	6	15	6	6 ^d^	6	6	6	6	6
RMF (5 Hz)	8 ^b^	15	8 ^b^	8 ^a^	9 ^b^	9 ^a^	9 ^b^	8 ^a^	8 ^a^
RMF (50 Hz)	9 ^a^	15	10 ^a^	8 ^a^	10 ^a^	9 ^a^	10 ^a^	8 ^a^	8 ^a^
Amoxicillin + Clavulanic Acid—β-lactams + inhibitor of β-lactamases									
Control	10	32	11	6 ^d^	11	13	13	15	9
RMF (5 Hz)	15 ^b^	32	17 ^b^	13 ^b^	19 ^a^	19 ^b^	20 ^a^	18 ^b^	17 ^a^
RMF (50 Hz)	16 ^a^	32	19 ^a^	17 ^a^	19 ^a^	20 ^a^	20 ^a^	19 ^a^	14 ^b^
Erythromycin—macrolides									
Control	25	27	6 ^c^	6 ^c^	6 ^c^	6 ^c^	6 ^c^	6 ^c^	10 ^c^
RMF (5 Hz)	25	29 ^a^	6 ^c^	6 ^c^	6 ^c^	6 ^c^	6	6 ^c^	12 ^ac^
RMF (50 Hz)	25	27	6 ^c^	6 ^c^	6	6 ^c^	6 ^c^	6 ^c^	10 ^c^
Clindamycin—lincosamides									
Control	27	25	6 ^c^	6 ^c^	6 ^c^	6 ^c^	6 ^c^	27	6 ^c^
RMF (5 Hz)	27	27 ^a^	6 ^c^	6 ^c^	6 ^c^	6 ^c^	6 ^c^	27	6 ^c^
RMF (50 Hz)	27	27 ^a^	6 ^c^	6 ^c^	6 ^c^	6 ^c^	6 ^c^	27	6 ^c^
Ciprofloxacin—fluoroquinolones									
Control	26 ^c^	28	6 ^c^	6 ^c^	6 ^c^	6 ^c^	6 ^c^	27 ^c^	6 ^c^
RMF (5 Hz)	26 ^c^	28	6 ^c^	6 ^c^	6 ^c^	6 ^c^	6 ^c^	27 ^c^	6 ^c^
RMF (50 Hz)	26 ^c^	28	6 ^c^	6 ^c^	6 ^c^	6 ^c^	6 ^c^	27 ^c^	6 ^c^
Tetracycline—tetracyclines									
Control	30	29	32	30	30	30	31	12 ^c^	15 ^c^
RMF (5 Hz)	30	29	36 ^a^	30	30	32 ^a^	31	12 ^c^	15 ^c^
RMF (50 Hz)	30	29	32	30	30	30	31	12 ^c^	15 ^c^
Gentamicin—aminoglycosides									
Control	25	23	27	23	26	25	26	23	6 ^c^
RMF (5 Hz)	25	23	27	25 ^a^	28 ^a^	27 ^a^	26	25 ^a^	6 ^c^
RMF (50 Hz)	25	23	27	23	26	25	26	23	6 ^c^
Teicoplanin—glycopeptide									
Control	18	20	18	19	19	19	18	16	18
RMF (5 Hz)	20 ^a^	22 ^a^	21 ^a^	21 ^a^	19	19	18	16	18
RMF (50 Hz)	20 ^a^	22 ^a^	21 ^a^	21 ^a^	21 ^a^	19	18	16	18

The differences in the diameter of the growth inhibition zones between three repetitions of the experiment did not exceed ±1 mm. The zones that are larger in comparison to the control are marked with letters: “^a^” means a larger zone, “^b^” a smaller one—as compared to each other. “^c^” indicates resistance according to the EUCAST clinical breakpoints (2021) [25]; “^d^” indicates a lack of sensitivity to clavulanic acid.

**Table 2:** MIC values (µg/mL) of β-lactam antibiotics for MRSA strains in control and RMF-exposed cultures.

Culture Conditions	Staphylococcal Strain								
	ATTC 33591	ATTC 6538	1	2	3	4	5	6	7
	Cefoxitin—β-Lactams—Methicillin Resistance Indicator								
Control	24	3	256	256	256	96	96	32	256
RMF (5 Hz)	16 ^a^	3	24 ^a^	6 ^a^	12 ^a^	16 ^a^	16 ^a^	16 ^a^	64 ^b^
RMF (50 Hz)	16 ^a^	3	24 ^a^	16 ^b^	12 ^a^	24 ^b^	24 ^b^	24 ^b^	48 ^a^
Amoxicillin—β-lactam									
Control	8	0.32	24	24^d^	24	16	16	6	32
RMF (5 Hz)	6 ^a^	0.32	6 ^a^	12 ^a^	4 ^b^	8 ^b^	6 ^b^	2 ^a^	24 ^a^
RMF (50 Hz)	4 ^a^	0.32	6 ^a^	12 ^a^	2 ^a^	6 ^a^	4 ^a^	3 ^b^	24 ^a^
Amoxicillin + Clavulanic Acid—β-lactams; aminopenicillins + inhibitor of β-lactamases									
Control	2	0.23	16	24 ^d^	16	12	12	3	16
RMF (5 Hz)	1.5 ^b^	0.23	4 ^a^	8 ^b^	3 ^a^	4 ^a^	4 ^b^	1 ^a^	12 ^a^
RMF (50 Hz)	0.75 ^a^	0.23	4 ^a^	4 ^a^	2 ^b^	4 ^a^	3 ^a^	2 ^b^	12 ^a^
		Erythromycin—macrolides							
Control	0.5	0.25	256 ^c^	256 ^c^	256 ^c^	256 ^c^	256 ^c^	256 ^c^	64
RMF (5 Hz)	0.38 ^a^	0.19 ^a^	256 ^c^	256 ^c^	256 ^c^	256 ^c^	256 ^c^	256 ^c^	48 ^a^
RMF (50 Hz)	0.5	0.25	256 ^c^	256 ^c^	256 ^c^	256 ^c^	256 ^c^	256 ^c^	64
		Clindamycin—lincosamides							
Control	0.125	0.94	256 ^c^	256 ^c^	256 ^c^	256 ^c^	256 ^c^	0.125	0.125
RMF (5 Hz)	0.94	0.64 ^a^	256 ^c^	256 ^c^	256 ^c^	256 ^c^	256 ^c^	0.125	0.125
RMF (50 Hz)	0.94	0.64 ^a^	256 ^c^	256 ^c^	256 ^c^	256 ^c^	256 ^c^	0.094 ^a^	0.125
Ciprofloxacin—fluoroquinolones									
Control	0.19	0.19	32 ^c^	32 ^c^	32 ^c^	4 ^c^	32 ^c^	0.125	32 ^c^
RMF (5 Hz)	0.19	0.19	32 ^c^	32 ^c^	32 ^c^	4 ^c^	32 ^c^	0.094 ^a^	32 ^c^
RMF (50 Hz)	0.19	0.19	32 ^c^	32 ^c^	32 ^c^	4 ^c^	32 ^c^	0.125	32 ^c^
		Tetracycline—tetracyclines							
Control	0.125	0.125	0.125	0.125	0.125	0.125	0.047	48	16
RMF (5 Hz)	0.125	0.125	0.094 ^a^	0.125	0.125	0.094 ^a^	0.047	48	16
RMF (50 Hz)	0.125	0.125	0.125	0.125	0.125	0.125	0.023 ^a^	48	16
		Gentamicin—aminoglycosides							
Control	0.25	0.19	0.125	0.38	0.19	0.19	0.19	0.125	256 ^c^
RMF (5 Hz)	0.19 ^a^	0.19	0.094 ^a^	0.25 ^a^	0.125 ^a^	0.125 ^a^	0.19	0.094 ^a^	256 ^c^
RMF (50 Hz)	0.19 ^a^	0.19	0.094 ^a^	0.25 ^a^	0.19	0.19	0.19	0.125	256 ^c^
Teicoplanin—glycopeptide									
Control	0.75	0.38	0.75	0.5	0.75	0.75	0.5	1.0	0.75
RMF (5 Hz)	0.38 ^a^	0.25 ^a^	0.5 ^a^	0.38 ^a^	0.5 ^a^	0.5 ^a^	0.5	1.0	0.75
RMF (50 Hz)	0.38 ^a^	0.25 ^a^	0.5 ^a^	0.38 ^a^	0.5 ^a^	0.75	0.38 ^a^	1.0	0.75

There were no differences in MIC values between three separate experiments. The zones that are larger in comparison to the control are marked with letters: “^a^” means a larger zone, “^b^” a smaller one—as compared to each other; “^c^” indicates resistance according to the EUCAST clinical breakpoints (2021) [25]; “^d^” indicates a lack of sensitivity to clavulanic acid.

**Table 3:** Mode of action of antibiotic classes selected for the study and related resistance mechanisms [35].

Antibiotic	Mode of Action	Resistance Mechanism
Β-lactams, Glycopeptides	Interference with cell wall synthesis	Reduced permeability Reduced affinity for antibiotic target Antibiotic hydrolysis
Macrolides Lincosamides	Inhibition of protein synthesis (binding to 50S ribosomal subunit)	Reduced affinity for antibiotic target Antibiotic hydrolysis Reduced uptake into cells
Aminoglycosides, Tetracyclines	Inhibition of protein synthesis (binding to 30S ribosomal subunit)	Inactivation of antibiotic by enzymatic modification Altered cell permeability Active efflux from cells
Fluoroquinolones	Inhibition of DNA synthesis	Alternation in antibiotic target Decreased cell permeability

**Table 4:** Character of antibiotics selected for the study depending on their charges.

Antibiotic	Character
Cefoxitin	anionic
Teicoplanin	
Erythromycin	cationic
Clindamycin	
Gentamicin	
Amoxicillin	zwitterionic
Tetracycline	
Ciprofloxacin	

## Data Availability

The data presented in this study are available on request from the corresponding author.

## Supplementary Materials

The following are available online at https://www.mdpi.com/article/10.3390/ijms222111551/s1.
