# Supplementary material for: The Effect of Rotating Magnetic Field on Susceptibility Profile of Methicillin-Resistant Staphylococcus aureus Strains Exposed to Activity of Different Groups of Antibiotics
Source: Int J Mol Sci. 2021 Oct 26;22(21):11551. doi: 10.3390/ijms222111551 (PMC8583794; doi:10.3390/ijms222111551)
Supplement: Supplementary file 1 [file ijms-22-11551-s001.zip › ijms-1414449-supplementary.pdf]

Supplementary Material

The Effect of Rotating Magnetic Field on Susceptibility Profile of Methicillin Resistant *Staphylococcus aureus* Strains Exposed to Activity of Different Groups of Antibiotics

Marta Woroszyło[1], Daria Ciecholewska-Juśko[1], Adam Junka[2,3*], Marcin Wardach[4], Grzegorz Chodaczek[5], Bartłomiej Dudek[6], Karol Fijałkowski[1*]

[1] Department of Microbiology and Biotechnology, Faculty of Biotechnology and Animal Husbandry, West Pomeranian University of Technology in Szczecin, Piastów 45, 70-311 Szczecin, Poland; marta.woroszylo@zut.edu.pl; daria.ciecholewska@zut.edu.pl; karol.fijalkowski@zut.edu.pl

[2] Department of Pharmaceutical Microbiology and Parasitology, Faculty of Pharmacy, Medical University of Wroclaw, Borowska 211a, 50-534 Wrocław, Poland; adam.junka@umed.wroc.pl

[3] Laboratory of Microbiology, Łukasiewicz Research Network–PORT Polish Center for Technology Development, 54-066 Wrocław, Poland; adam.junka@port.lukasiewicz.gov.pl

[4] Faculty of Electrical Engineering, West Pomeranian University of Technology in Szczecin, Sikorskiego 37, 70-313 Szczecin, Poland; marcin.wardach@zut.edu.pl

[5] Laboratory of Confocal Microscopy, Łukasiewicz Research Network - PORT Polish Center for Technology Development, Stabłowicka 147, 54-066 Wrocław, Poland; grzegorz.chodaczek@port.pl

[6] Department of Microbiology, Institute of Genetics and Microbiology, University of Wrocław, Stanisława Przybyszewskiego 63, 51-148 Wrocław, Poland; bartlomiej.dudek@uwr.edu.pl

***Correspondence:**
KF: karol.fijalkowski@zut.edu.pl; + 48 91-449-6714;
AJ: adam.junka@umed.wroc.pl; +48 +48-88-922-93-41.

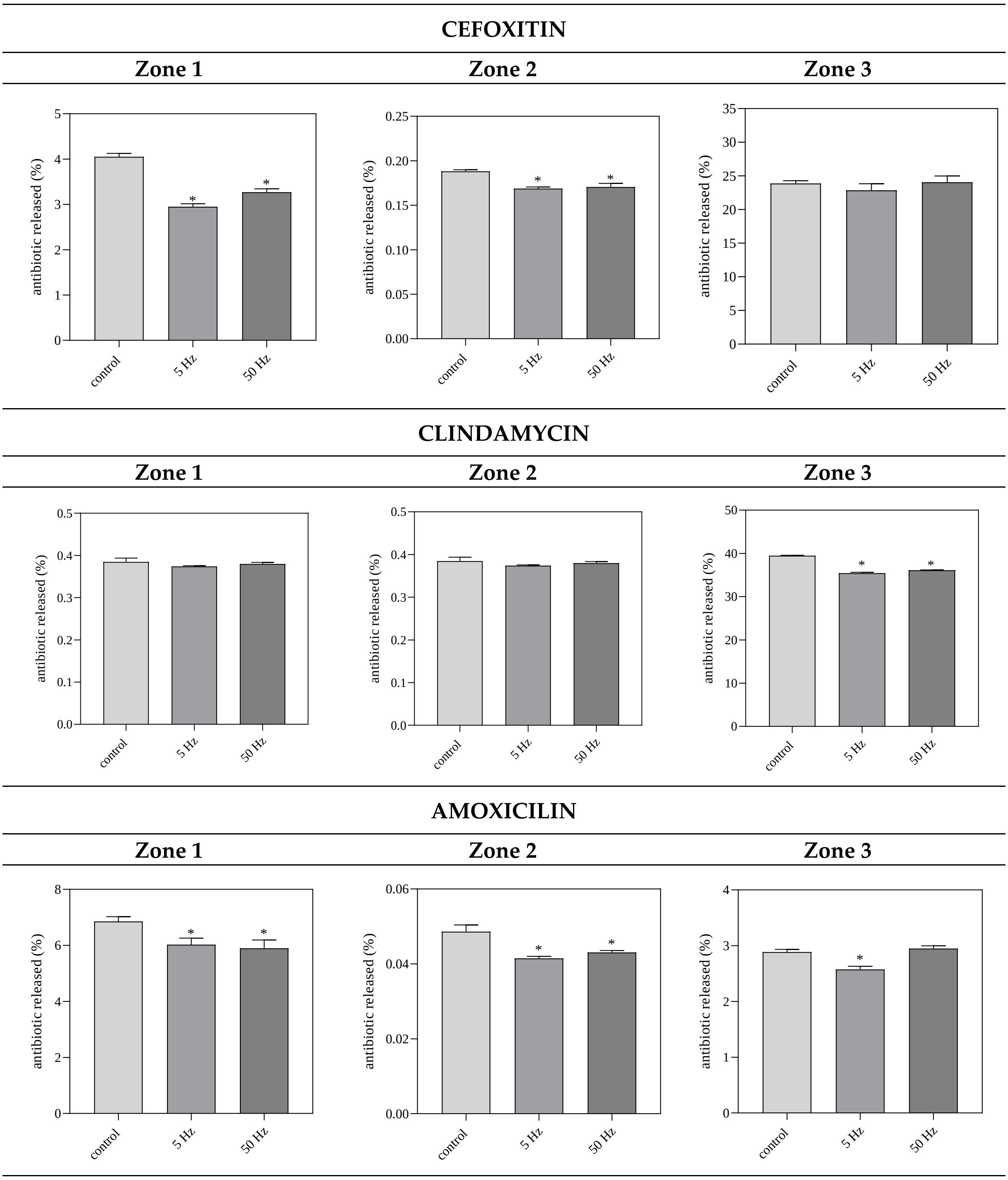
CEFOXITIN
Zone 1
Zone 2
Zone 3
antibiotic released (%)
control
5 Hz
50 Hz
CLINDAMYCIN
Zone 1
Zone 2
Zone 3
antibiotic released (%)
control
5 Hz
50 Hz
AMOXICILIN
Zone 1
Zone 2
Zone 3
antibiotic released (%)
control
5 Hz
50 Hz

TETRACYCLINE		
**Zone 1**	**Zone 2**	**Zone 3**
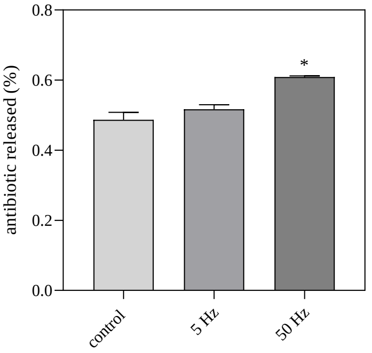	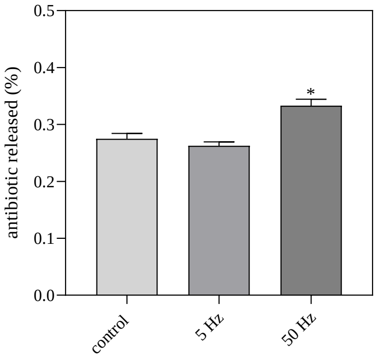	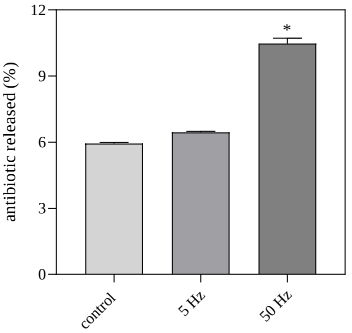
**GENTAMYCIN**		
**Zone 1**	**Zone 2**	**Zone 3**
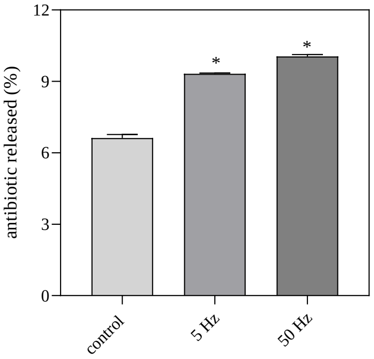	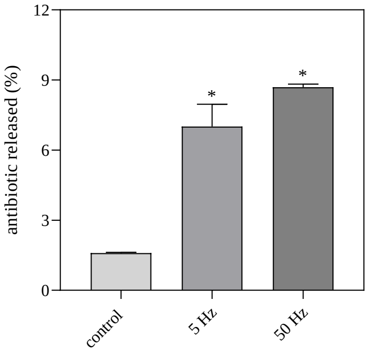	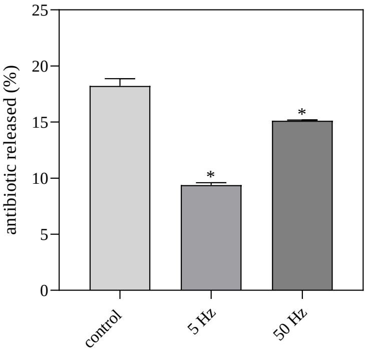
**ERYTHROMYCIN**		
**Zone 1**	**Zone 2**	**Zone 3**
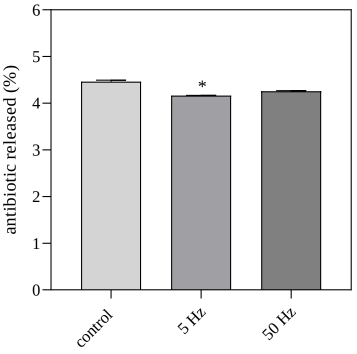	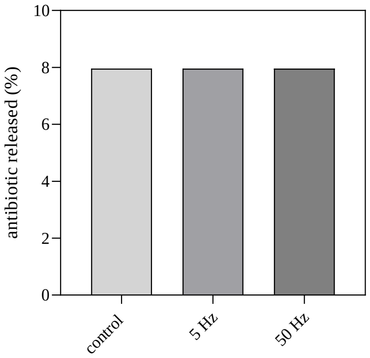	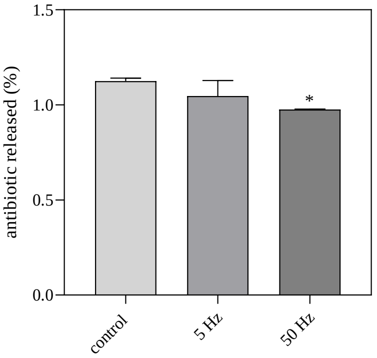

CIPROFLOXACIN

Zone 1	Zone 2	Zone 3

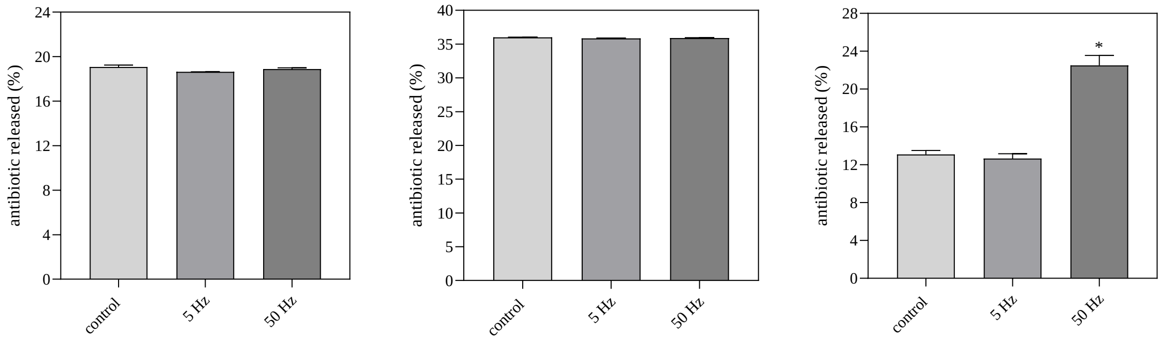

Figure S1. Percentage of antibiotic released from the paper disc during 120 min in control and RMF-exposed (5/50 Hz) settings. The results are presented as a mean ± SEM calculated using six values (three from each biological replicate). * - indicates statistical differences (p<0.05) between control and RMF-exposed settings.

(a) (b)

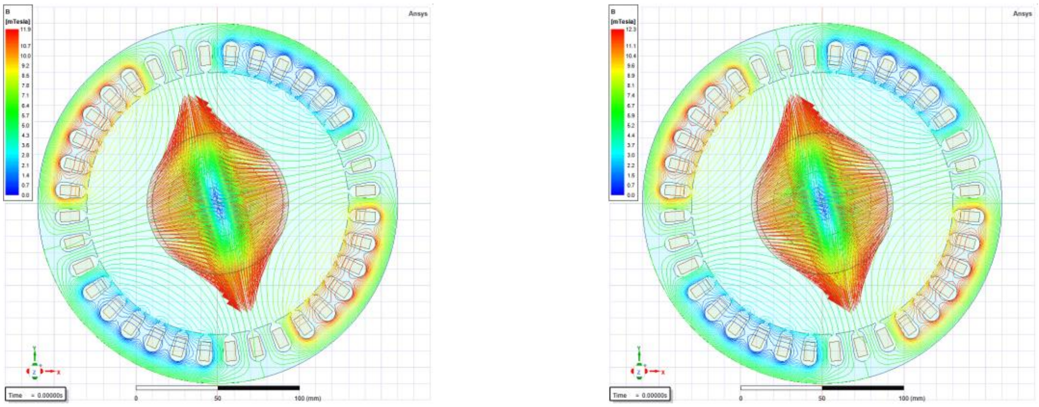

Figure S2. Visualization of generated magnetic flux rotation around the stator inside the RMF reactor chamber at (**a**) 5 Hz and (**b**) 50 Hz (please click the underlined RMF frequencies to watch the simulation).
